# DHEA and Cortisol in Rainbow Trout (*Oncorhynchus mykiss*): Effect of Sex, Sexual Maturity, and Acute Stress Exposure

**DOI:** 10.3390/ani15182710

**Published:** 2025-09-16

**Authors:** Andrea Meloni, Martina Bortoletti, Elena Negrato, Elisa Fonsatti, Giuseppe Radaelli, Daniela Bertotto

**Affiliations:** Department of Comparative Biomedicine and Food Science (BCA), University of Padova, Viale dell’Università 16, Legnaro, I-35020 Padova, Italy; andrea.meloni@phd.unipd.it (A.M.); elena.negrato@unipd.it (E.N.); elisa.fonsatti@unipd.it (E.F.); giuseppe.radaelli@unipd.it (G.R.); daniela.bertotto@unipd.it (D.B.)

**Keywords:** DHEA, cortisol, stress biomarkers, rainbow trout (*Oncorhynchus mykiss*), gonadal histology

## Abstract

Glucocorticoids like cortisol are widely used to assess stress in fish, but their interpretation can be limited. Incorporating dehydroepiandrosterone (DHEA), a hormone with anti-glucocorticoid and neuroprotective effects, may provide a broader understanding of hypothalamic–pituitary–inter-renal (HPI) axis activity. As DHEA data in fish remain scarce, this study examined its role in rainbow trout (*Oncorhynchus mykiss*) subjected to acute stress (30 min confinement), considering sex and sexual maturity. Cortisol and DHEA were quantified by optimized radioimmunoassay (RIA) in serum, muscle, fin, and scales, and gonadal histology was performed to confirm reproductive status. Both hormones were successfully measured in all matrices, with serum DHEA levels notably higher than those reported to date in fish. As expected, serum cortisol increased markedly after stress and correlated with levels in muscle and fin but not in scales, which appears to reflect chronic rather than acute exposure. In contrast, serum DHEA showed no stress-induced changes and only minor sex- and maturity-related differences, although alternative matrices displayed variable patterns, particularly in muscle and fin. The cortisol/DHEA ratio in serum mirrored cortisol dynamics, suggesting limited utility for acute stress assessment, while tissue-specific DHEA variation may integrate longer-term influences. Further research is needed to clarify the role of DHEA under chronic stress and its potential origins in inter-renal tissue, gonads, or the brain.

## Simple Summary

Cortisol is widely used to assess stress in fish, but other hormones like dehydroepiandrosterone (DHEA), known for its anti-glucocorticoid and protective effects, may offer a broader view. This study examined cortisol and DHEA levels in rainbow trout (*Oncorhynchus mykiss)* after 30 min of confinement, considering sex and sexual maturity. Hormones were analyzed in serum, muscle, fin, and scales, and gonadal histology was used to confirm maturity. Both hormones were quantified in all tissues, with serum DHEA levels higher than those reported to date in fish. Cortisol increased sharply after stress and correlated with muscle and fin levels, but not with scales. DHEA in serum did not change with acute stress and showed only minor differences related to sex and maturity, though tissue-specific variation occurred in muscle and fin, unaffecting scales. The cortisol/DHEA ratio in serum followed cortisol patterns, limiting its value for acute stress assessment. Further studies should investigate the physiological functions of DHEA under chronic stress and its potential production sites in inter-renal tissue, gonads, or the brain.

## 1. Introduction

Dehydroepiandrosterone (DHEA) is a steroid historically considered to act only as a pro-hormone, but it has proven useful for assessing chronic stress levels in humans [1]. In mammals, it is synthesized in steroidogenic tissues (adrenal glands, gonads, placenta, nervous system), from pregnenolone via the Δ5 pathway, mediated by P450c17 (CYP17A) [2,3]. Sharing the same precursor as cortisol, its secretion depends at least in part on adrenocorticotropic hormone (ACTH) and reflects activation of the hypothalamic–pituitary–adrenal (HPA) axis [2]. For this reason, in humans and animals, DHEA and its sulfate form (DHEA-S) are mainly associated with stress responses, but it has been observed that they are also influenced by sex, reproductive status, age, health, and season. For instance, during the breeding season, red squirrels show higher adrenal than gonadal DHEA levels, whereas in squirrel monkeys and male killer whales during summer, the testes become the main source of DHEA [4,5], a pattern that could be related to rises in hormone levels due to male social challenges as suggested in the context of the challenge hypothesis [6]. These examples highlight the importance of identifying the origin of DHEA and the species-specific factors influencing its levels, especially if this hormone is to be used as a stress biomarker in animals. DHEA also plays a reproductive role, being a precursor of sex steroids (androgens, estrogens) and possibly protecting gonadal tissues from cortisol’s adverse effects via 11β-HSD2–mediated conversion of cortisol to cortisone, its inactive form [7,8,9]. Circulating levels of DHEA have been shown to vary in association with gonadal development and reproductive activity across different species [2,3,10]. Beyond reproduction, DHEA exerts neuroprotective, antioxidant, anti-inflammatory, and anti-glucocorticoid effects, mitigating the impact of stress [3,11,12,13].

Cortisol has traditionally been the primary biomarker for stress, but its role is now being redefined, because its interpretation is complicated by several factors. In fact, it is secreted not only in response to negative stressors but also to positive stimuli such as arousal or excitement [2,14]. Cortisol levels are strongly influenced by handling and blood collection during sampling, which themselves can trigger acute stress response and confound results [15,16]. Moreover, serum or plasma concentrations provide only a short-term snapshot and have been shown to be unreliable indicators of chronic stress [17,18]. Due to these limitations, alternative biological matrices have been explored over the past two decades in both terrestrial and aquatic species [16,17,19]. These matrices are often less invasive, as in the case of saliva and mucus [16,20], less affected by sampling-induced stress due to the time required for hormones to diffuse from blood to tissues, and can reflect prolonged, chronic stress, as seen in hair and claws in mammals, feathers in birds, and scales in fish, that accumulate cortisol over time [16,20,21,22]. In addition to being linked to stress conditions, cortisol, like DHEA, exhibits variations in concentration related to reproductive status and gonadal maturation [23,24,25,26].

Given their opposing actions, the cortisol/DHEA ratio has been proposed as a more reliable indicator of HPA axis activity than either hormone alone [2,17,27]. In humans, a high ratio is associated with prolonged stress, illness, and anxiety, while in animals, it has been linked to resilience and allostatic load [3,28]. Simultaneous evaluation of both hormones, across traditional and alternative matrices, may therefore provide a more comprehensive assessment of stress and welfare.

Although data on non-mammals are scarce, DHEA has been detected in teleosts, where it appears to be related to chronic stress exposure [29,30,31,32]. Protocols have recently been developed for its quantification (or for its sulphated form) together with cortisol and cortisone in various matrices (e.g., blood, skin, and scales), although the effects of sex, maturity, and acute stress remain poorly explored.

In order to investigate these aspects, the present study aimed to examine DHEA levels in rainbow trout (*Oncorhynchus mykiss*), focusing on the potential effect of acute stress exposure, sex, and maturity. The hormone level was assessed in animals of the same age, both males and females, with different gonadal maturity, exposed or not to an acute stress produced by confinement, in order to obtain information on the potential influence of these factors. Given their relationship with each other and with stress, DHEA was assessed together with cortisol in serum and other biological matrices, such as muscle, fin and scales. The alternative matrices were used in order to identify matrices that are less sensitive to sampling stress and capable of detecting concentrations related to chronic or pre-sampling conditions. The development of analytical protocols for DHEA quantification, in both traditional and alternative matrices, alongside cortisol measurement, aimed to offer new tools for hormone assessment and a more comprehensive understanding of the stress response and HPI axis activation over both short and long terms in this species. The contemporary measurement of DHEA and cortisol has allowed to explore whether their ratio is influenced by sex, maturity stage, and acute stress exposure, and whether it could also serve as a reliable stress biomarker in fish, as in humans and other mammals, across different biological matrices.

Gonadal histology was conducted to confirm sex and maturity stages, ensuring accurate interpretation of hormone levels in relation to the reproductive status.

Rainbow trout was chosen as the model species for this study because it is highly relevant for aquaculture in Italy and Europe [33] and this guaranteed the simultaneous availability of both males and females of the same age but different gonadal maturity on farm, allowing for the joint assessment of these factors. In addition, the species has served as a model for over a century in fields such as carcinogenesis, toxicology, immunology, ecology, physiology, and nutrition and this provides a wide availability of basic biological data [34].

## 2. Materials and Methods

### 2.1. Sampling

Three-year-old rainbow trout (*Oncorhynchus mykiss*) were sampled from a private commercial freshwater aquaculture farm in northern Italy during the spawning season. To include both sexes and different maturity stages at the same age, a total of 96 fish (mean body weight in Table 1) were collected from breeding stocks. Following standard farm practices, the fish were maintained in two sex-separated tanks under identical conditions in outdoor raceways supplied with spring water. Throughout the year, water temperatures naturally ranged from 2 to 20 °C, dissolved oxygen levels remained above 90% saturation, and stocking density was maintained at 30 kg/m^3^.

The experimental design comprised four groups based on sex (male or female) and sexual maturity (mature or immature), with 24 individuals per group. At sampling, fish were handled according to routine slaughter procedures and rapidly confined at the end of the tanks. For each group, 12 individuals were captured and euthanized by percussive stunning within 5 min of confinement, serving as non-stressed controls.

The remaining 12 fish were subjected to acute stress by being kept confined in the tank for 30 min prior to slaughter, simulating farm pre-slaughter conditions. Although pre-slaughter waiting times in the tanks under farming practices may vary, the 30 min duration in the present study was selected based on scientific evidence indicating that this period is sufficient to elicit a significant rise in cortisol concentration in both blood and fin tissues of rainbow trout under acute stress [35,36,37]. This final design resulted in eight experimental groups (*n* = 12 per group), based on the combination of sex, maturity status, and stress conditions (control or stressed), as detailed in Table 1.

### 2.2. Matrices

Blood, muscle, fin, and scales were collected from each fish to assess hormone concentrations. Blood was withdrawn from the caudal vein using 2.5 mL syringes fitted with a 22G needle, stored at 4 °C overnight before centrifugation (4 °C, 5000× *g*, for 10 min), to isolate serum, which was then stored at −20 °C until analysis. Scales were collected by scraping the surface of the fish using sterile plastic sticks and forceps, respectively. The upper lobe of the caudal fin was excised with scissors, and a 1 cm^3^ sample of skinless muscle tissue was collected from the upper half of the caudal peduncle on the left side of the fish using a sterile scalpel. All tissue samples were stored at −20 °C until further analysis.

### 2.3. Hormones Extraction and Radioimmunoassays (RIAs)

For cortisol and DHEA assessment, a solid specific microtiter radioimmunoassay (RIA) was used as described in Bertotto et al. [38], with modifications in sample homogenization and specific antiserum incubations. Briefly, a 96-well plate (Optiplate, Perkin Elmer Life Sciences, Shelton, CT, USA) was coated with anti-rabbit γ-globulin and incubated for 2 h at 37 °C with the specific antiserum solutions for cortisol or DHEA (Centro Medico Diagnostico Emilia, Bologna, Italy). Standards, controls, extracts, and 3H tracers were then added and, after overnight incubation at 4 °C, the plate was washed with PBS, filled with scintillation cocktail (Microscint 20, Perkin Elmer Life Sciences) and counted on a counter (Perkin Elmer Life Sciences). Antiserums cross-reactivities were as follows: (1) Anti-Cortisol: cortisol 100%, prednisolone 44.3%, 11-deoxycortisol 13.9%, cortisone 4.95%, corticosterone 3.5%, prednisone 2.7%, others <1%; (2) Anti-DHEA: DHEA 100%, 5α-androsten-3β,17β-diol 9.2, epiandrosteron 2.8%, others <1%. Before RIAs, steroids were extracted from the matrices after cleaning and weighing samples (muscle: 50 ± 3 mg; fin: 30 ± 3 mg; scales: 25 ± 3 mg) were extracted with 8 mL diethyl ether. Before the steroids’ extraction, scales and fin were pre-washed overnight in isopropanol and then dried under nitrogen current. Given the limited amount of material, scales from two individuals of the same group were pooled. The assays’ performance was validated by parallelism, extractive yield, and inter/intra-assay precision tests, which were carried out in all matrices. The sensitivity of the assay was defined as the dose of hormone at 95% binding (B/B0) and was 1.5 pg/well.

### 2.4. Gonad Histology

To assess sexual maturity, gonads were collected and evaluated by classical histological preparation and microscopic observation. Briefly, ovaries and testes were fixed at 4 °C overnight in formalin at 10% in phosphate-buffered saline (PBS, 0.1 M, pH 7.4), after which they were washed in PBS and stored in 70% ethanol at 4 °C until use. The samples were then dehydrated through a series of graded ethanol, cleared in xylene, and embedded in paraffin wax. Five μm thick consecutive sections were cut using a microtome RM2125 RTS (Leica Biosystems, Wetzlar, Germany) and stained with Meyers’s hematoxylin and eosin. Images were acquired using a Zeiss Axio Imager M2 microscope (Zeiss, Oberkochen, Germany). Maturity stages were determined following Santos et al. [39]. Based on this classification, females were categorized into five stages (1–5), ranging from early developmental phases with previtellogenic oocytes (stage 1–early development) to advanced postovulatory stages characterized by residual oocytes and predominant postovulatory follicles (stage 5). Males were categorized into four stages (0–4), from the most immature, showing only spermatogonia (stage 0), to spent (stage 4), passing through the stage 3 with predominantly mature spermatozoa.

### 2.5. Statistical Analysis

All analyses were performed in R (version 4.5.0, [40]).

Prior to analysis, data were assessed for normality and homoscedasticity using the Shapiro–Wilk test and Levene’s test, followed by a visual inspection of the residuals and the distribution shape to assess the distributional assumptions. The factors considered were sex, sexual maturity, and stress, with weight included as a covariate to analyze cortisol, DHEA, and their ratio in serum, muscle, fin, and scales. Data were log-transformed where necessary to achieve normality. For data meeting the normality assumption, a multi-way ANOVA was conducted, followed by Tukey’s post hoc test to address multiple comparisons. As a non-parametric test, the Kruskal–Wallis test was used to detect differences among fixed effects, and pairwise Wilcoxon rank-sum tests with Bonferroni correction were applied for pairwise comparisons when significant effects were observed. Correlations between variables were assessed using Pearson’s correlation test. Results are expressed as hormone concentrations in ng/mL or ng/g ± SE, unless otherwise specified.

A significance level of *p* < 0.05 was adopted for all tests.

## 3. Results

### 3.1. Fish

Group characteristics in terms of sex, maturity, and total body weight are reported in Table 1. Immature fish were significantly heavier than the mature ones independently of the sex and stress status (Table 1).

### 3.2. RIAs Validation

Cortisol and DHEA RIAs were successfully validated for all the matrices, and the test parameters are reported in Table 2. The relationships between cortisol and DHEA in the different matrices and their respective standard curves, determined through linear regressions, were linear, with correlation coefficients R^2^ always over 0.99. Extraction yield was 80% or higher for cortisol and always higher than 70% for DHEA. Intra-assay and inter-assay values were lower than 8.73 and 7.54, respectively, attesting to the assays’ good precision and repeatability (Table 2).

### 3.3. Hormone Concentration in the Serum

#### 3.3.1. Cortisol

**Figure 1:**
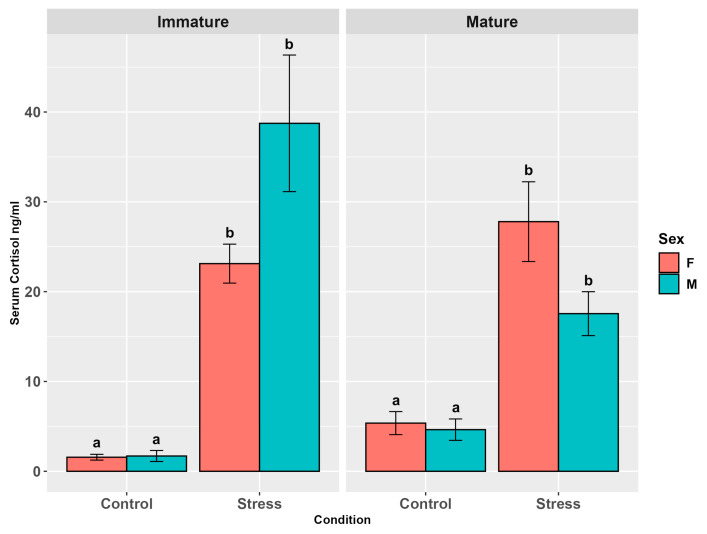
Serum cortisol levels (ng/mL ± SE) in the experimental groups by stress, sex, and maturity. Statistical analysis was performed using a multi-way ANOVA followed by a Tukey post hoc test. Different letters indicate statistical differences among the groups (*p* < 0.05).

Cortisol levels in serum increased significantly in the trout of the stressed group compared to the controls (*p* < 0.001, Table S1), while no difference was observed between the sexes (*p* = 0.81). Maturity showed a significant effect (*p* < 0.05) that was not confirmed in the pairwise comparison (*p* = 0.91). The interaction between maturity and stress was highly significant (*p* < 0.0001) and this result was confirmed by all the pairwise comparisons among the stressed vs. control groups. Cortisol levels in stressed animals showed tendential differences among males, with higher levels in immature individuals (*p* = 0.07, Table S2a). The interaction between maturity and sex, sex and stress, and among the three factors were not significant (*p* = 0.32; *p* = 0.39; *p* = 0.15). However, the pairwise comparisons highlighted higher cortisol levels in all the stressed groups than in the controls (*p* < 0.0001, Figure 1).

#### 3.3.2. DHEA

**Figure 2:**
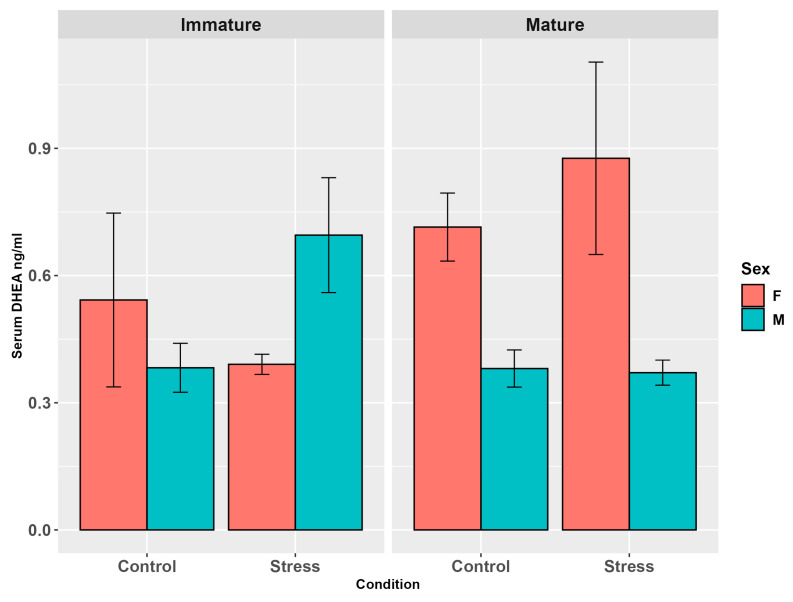
Serum DHEA levels (ng/mL ± SE) in the experimental groups by stress, sex, and maturity. Statistical analysis was performed using a multi-way ANOVA followed by Tukey post hoc test. Different letters indicate statistical differences among the groups (*p* < 0.05). The absence of different letters indicates that there are no significant differences between the hormone levels of the different groups.

DHEA concentrations in the serum did not exhibit any differences related to stress (*p* = 0.26), maturity (*p* = 0.09), or interaction among the three factors (*p* = 0.24, Figure 2, Table S2b). Sex showed a marginal influence (*p* = 0.05), with females higher than males at the pairwise comparison (*p* < 0.05). The interaction between maturity and sex was highly significant (*p* < 0.0001), with mature females exhibiting significantly higher values compared to immature females (*p* < 0.05) and mature males (*p* < 0.0001, Table S2b).

#### 3.3.3. Cortisol/DHEA Ratio

**Figure 3:**
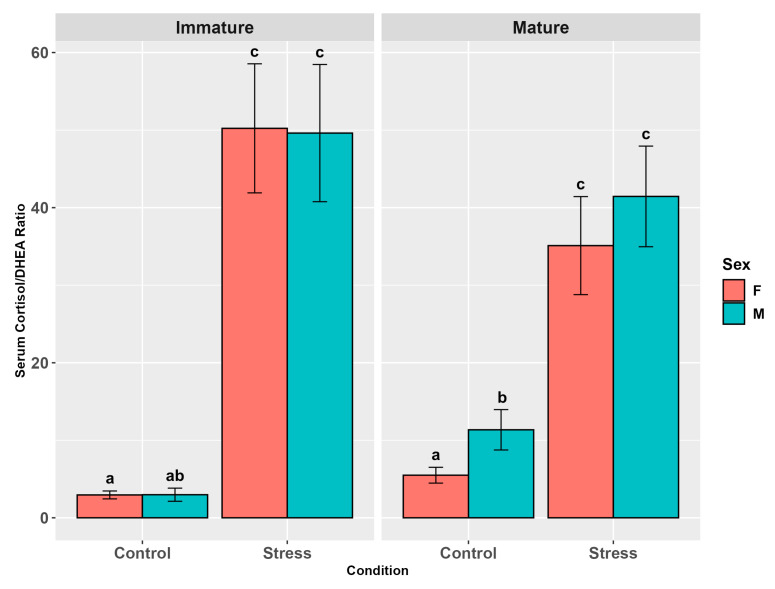
Serum cortisol/DHEA ratio in the experimental groups by stress, sex, and maturity. Statistical analysis was performed using a multi-way ANOVA followed by Tukey post hoc test. Different letters indicate statistical differences among the groups (*p* < 0.05).

The serum cortisol/DHEA ratio followed a consistent cortisol pattern with higher levels in stressed animals (*p* < 0.0001, Table S1c) and a significant influence of maturity (*p* < 0.05, Table S1c), the latter not confirmed by the pairwise comparison (*p* = 0.74). The ratio also showed a significant effect of sex (*p* < 0.05, Table S1c), with values tending to be higher in males than in females (*p* = 0.07, Figure 3, Table S1c).

The interaction among the three factors, although not significant overall (*p* = 0.43), in pairwise comparisons showed higher values in stressed animals than in controls (*p* < 0.05; *p* < 0.001; *p* < 0.0001) and, among controls, in mature males than in mature and immature females (*p* < 0.05, Figure 3, Table S2c). The interaction between maturity and stress was also close to the significance threshold (*p* = 0.053). Relative pairwise comparisons showed higher values in stressed mature and immature animals than in immature controls (*p* < 0.0001) and in stressed immature individuals than in mature controls (*p* < 0.0001).

### 3.4. Hormone Concentration in Alternative Matrices

RIAs successfully assessed cortisol and DHEA concentrations in all the alternative matrices tested, such as muscle, fins, and scales.

#### 3.4.1. Cortisol

**Figure 4:**
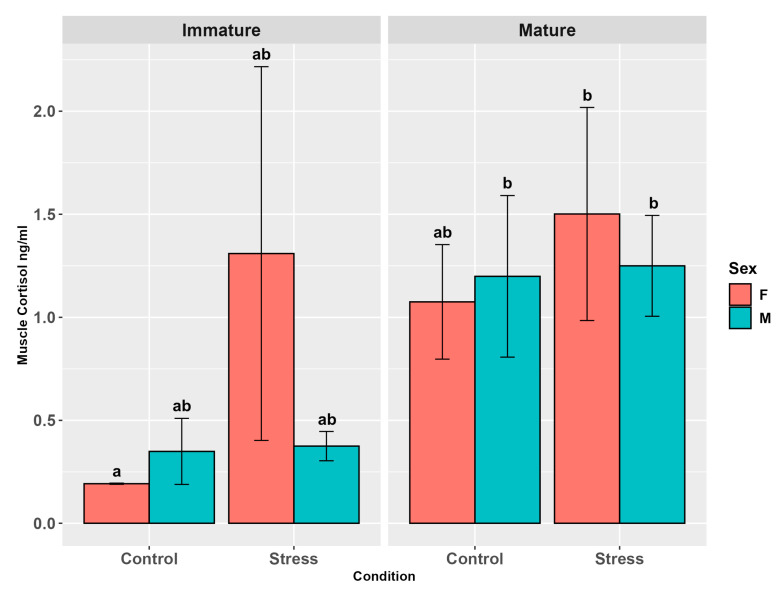
Muscle cortisol levels (ng/g ± SE) in the experimental groups by stress, sex, and maturity. Statistical analysis was performed using a multi-way ANOVA for each matrix followed by a Tukey post hoc test. Different letters indicate statistical differences among the groups (*p* < 0.05).

In muscle, stressed animals exhibited higher levels of cortisol than controls (*p* < 0.05). Also, maturity showed a significant effect (*p* < 0.0001), with mature individuals presenting higher levels than immature ones (*p* < 0.01, Table S1a), while no influence of sex was observed (*p* = 0.87, Table S1a). The interaction between sex and stress (*p* = 0.19), maturity and sex (*p* = 0.54), maturity and stress (*p* = 0.18), and among all the three factors (*p* = 0.25) did not show any significant effects (Figure 4, Table S2a). However, relative pairwise comparisons for sex and stress evidenced differences between females, with higher levels in stressed individuals (*p* < 0.05), and for sex and maturity, between males, with mature individual levels higher than the immature ones (*p* < 0.05).

The comparisons between maturity and stress showed lower levels in immature controls than in mature controls (*p* < 0.01) and stressed mature (*p* < 0.001) and immature individuals (*p* < 0.05). Three-factor pairwise comparisons evidenced higher levels in stressed mature males (*p* < 0.01) and females (*p* < 0.01) and in mature male controls (*p* < 0.05) than in immature female controls (Figure 4, Table S2a).

**Figure 5:**
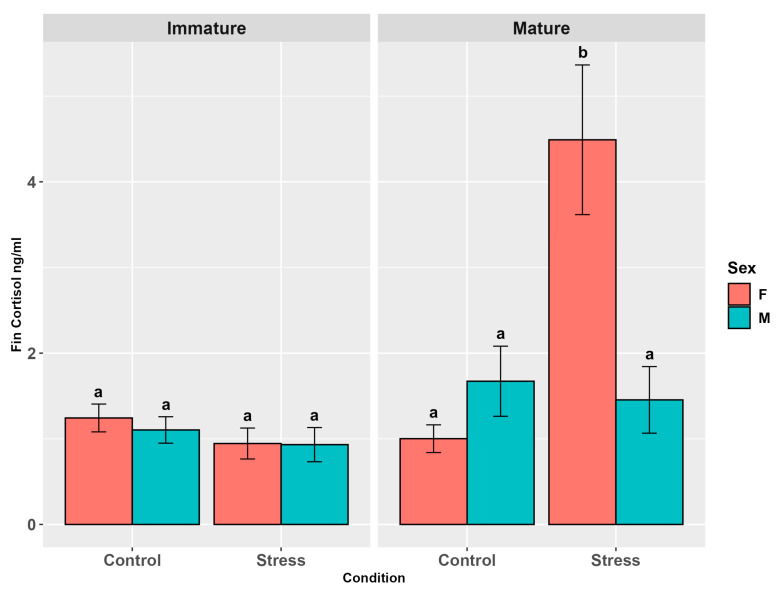
Fin cortisol levels (ng/g ± SE) in the experimental groups by stress, sex, and maturity. Statistical analysis was performed using a multi-way ANOVA followed by a Tukey post hoc test. Different letters indicate statistical differences among the groups (*p* < 0.05).

Fin cortisol showed no influence of stress (*p* = 0.23, Table S1a) and the sex effect was close to the significant threshold (*p* = 0.052, Table S1a). Maturity significantly influenced cortisol levels (*p* < 0.001), with mature individuals showing higher levels than immature ones (*p* < 0.04, Table S1a). The interaction among the three factors (*p* < 0.01, Figure 5) and all those between two factors, except maturity per sex (*p* = 0.4), were significant (*p* < 0.05), highlighting the relatively higher levels across all sub-groups that include stressed mature females (*p* < 0.05, Figure 5, Table S2a).

**Figure 6:**
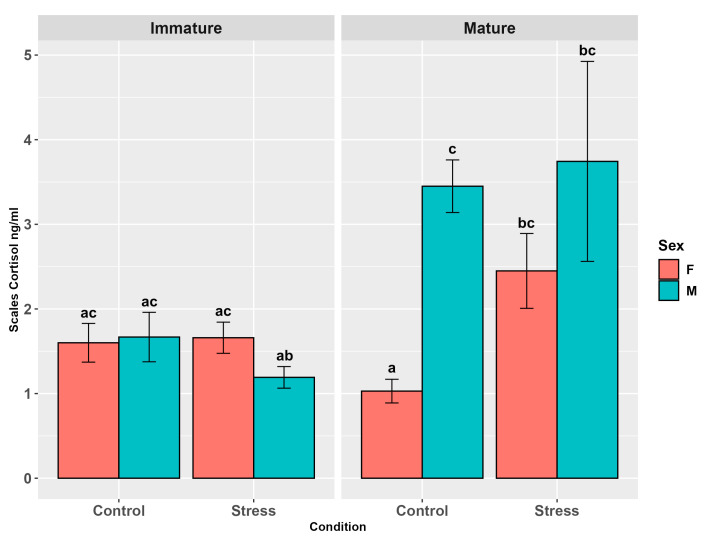
Scale cortisol levels (ng/g ± SE) in the experimental groups by stress, sex, and maturity. Statistical analysis was performed using a multi-way ANOVA followed by a Tukey post hoc test. Different letters indicate statistical differences among the groups (*p* < 0.05).

Scale cortisol showed only a tendential influence of stress (*p* = 0.07), but a significant effect of maturity (*p* < 0.01, Table S1a), though not confirmed by the pairwise comparisons (stress, *p* = 0.28; maturity, *p* = 0.16, Table S1a); however, sex differences were significant (*p* < 0.05), with males showing higher levels. All the two-factor interactions were significant (*p* < 0.05, *p* < 0.001) and the pairwise comparisons highlighted higher scale cortisol levels in mature males than in both mature females (*p* < 0.001) and immature males (*p* < 0.05), and female controls showing lower levels than male controls (*p* < 0.01), stressed males (*p* < 0.05), and stressed females (*p* < 0.05). The interaction among the three factors was not significant (*p* = 0.45, Figure 6, Table S2a), but differences among groups were detected in the pairwise comparisons. Among mature trout, female controls showed lower levels than male controls (*p* < 0.001) and stressed males (*p* < 0.01) and females (*p* < 0.05). In addition, mature male control levels were higher than those of the stressed immature males (*p* < 0.05).

#### 3.4.2. DHEA

**Figure 7:**
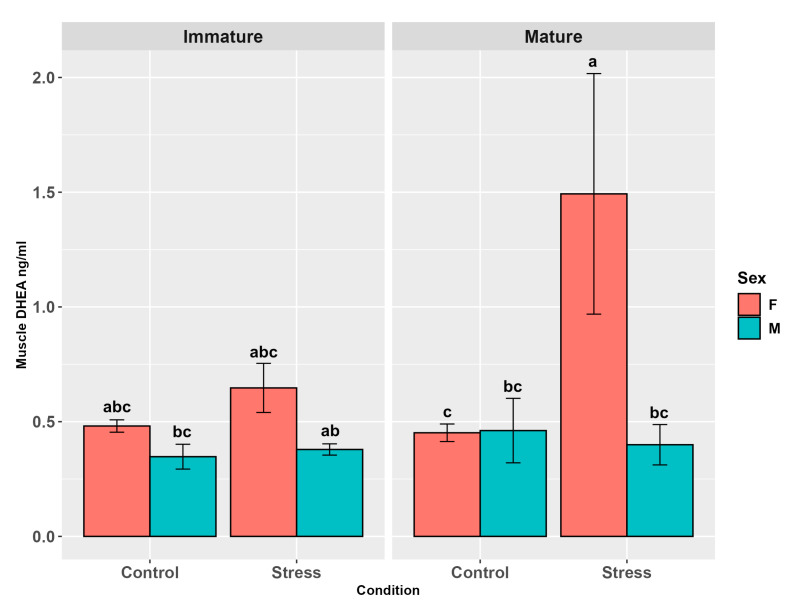
Muscle DHEA levels (ng/g ± SE) in the experimental groups by stress, sex, and maturity. Statistical analysis was performed using a multi-way ANOVA followed by Tukey post hoc test. Different letters indicate statistical differences among the groups (*p* < 0.05).

Muscle DHEA levels were influenced by stress (*p* < 0.05) and sex (*p* < 0.0001) but not by maturity (*p* = 0.3, Table S1b). Stressed animals showed higher levels than controls (*p* < 0.05) and females higher levels than males (*p* < 0.0001, Table S1a). The interaction between sex and stress (*p* = 0.043) highlighted higher levels in stressed females than in female and male controls, and in stressed males (*p* < 0.05; *p* < 0.001; *p* < 0.001). Interactions between maturity and sex and maturity and stress were not significant (*p* = 0.25; *p* = 0.38) but the pairwise comparisons in the latter showed higher levels in mature females than in mature males (*p* < 0.001). The three-way interaction showed a tendency (*p* = 0.06, Figure 7, Table S2b), in the pairwise comparisons, towards higher DHEA levels in stressed mature females than in both mature female control (*p* < 0.05, Table S2b) and mature male control (*p* < 0.01), immature male controls (*p* < 0.01), and stressed mature males (*p* < 0.001). In addition, stressed immature male levels were higher than in immature male controls (*p* < 0.05).

**Figure 8:**
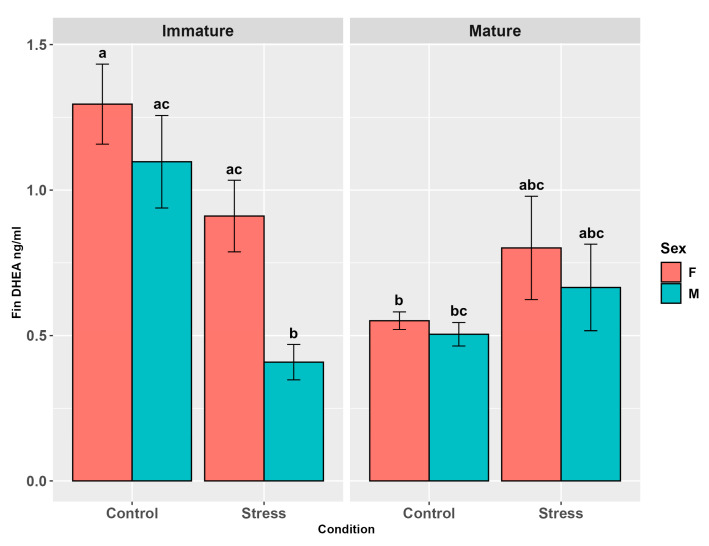
Fin DHEA levels (ng/g ± SE) in the experimental groups by stress, sex, and maturity. Statistical analysis was performed using a multi-way ANOVA followed by a Tukey post hoc test. Different letters indicate statistical differences among the groups (*p* < 0.05).

Fin DHEA was influenced by stress (*p* < 0.05), sex (*p* < 0.01), and maturity (*p* < 0.01, Table S1b). Non-stressed individuals (controls) and females showed higher levels than stressed animals and males, respectively (*p* < 0.05; *p* < 0.01, Table S1b), while maturity pairwise comparisons did not show any differences among groups (*p* = 0.37). The significant interaction between maturity and sex (*p* < 0.05) revealed higher DHEA levels in immature females than in immature males (*p* < 0.01). Also, the interaction between maturity and stress was highly significant (*p* < 0.001), showing higher levels in immature controls than in mature controls and stressed immature individuals (*p* < 0.05; *p* < 0.001). Both female controls and stressed females showed higher levels than stressed males (*p* < 0.001; *p* < 0.05), though the interaction between sex and stress was not significant (*p* = 0.5). The three-factor interaction did not show any significant difference (*p* = 0.34, Figure 8, Table S2b) but the pairwise comparisons highlighted higher values of DHEA in fin of immature female controls than in mature female (*p* = 0.046) and male controls (*p* < 0.05) and stressed immature males (*p* < 0.0001). The latter also exhibited lower levels than immature male controls (*p* < 0.01) and stressed immature females (*p* < 0.05, Figure 8, Table S2b).

**Figure 9:**
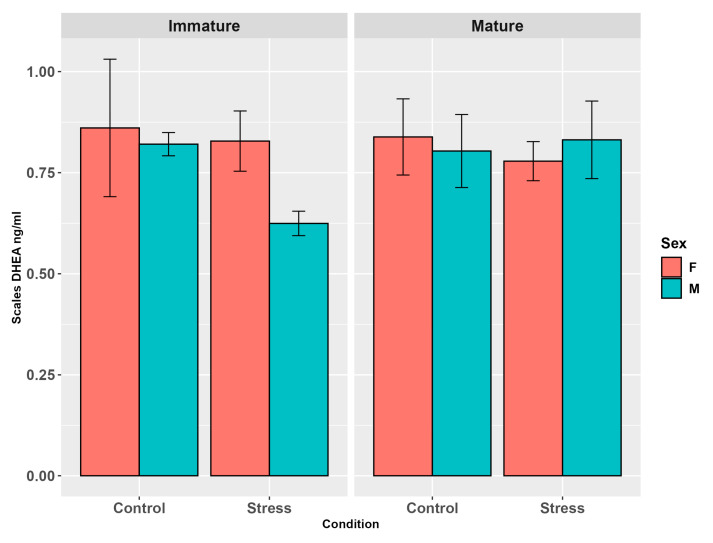
Scale DHEA levels (ng/g ± SE) in the experimental groups by stress, sex, and maturity. Statistical analysis was performed using a Kruskal–Wallis test followed by Wilcoxon rank sum test. Different letters indicate statistical differences among the groups (*p* < 0.05). The absence of different letters indicates that there are no significant differences between the hormone levels of the different groups.

Scale DHEA levels showed no influence of stress (*p* = 0.46, Table S1b), sex (*p* = 0.47, Table S1b), or maturity (*p* = 0.40, Table S1b). No significant effects were found in the three-factor interaction (*p* = 0.55, Figure 9, Table S2b) or in the two-way interactions: sex and stress (*p* = 0.48), maturity and sex (*p* = 0.68), maturity and stress (*p* = 0.58).

#### 3.4.3. Cortisol/DHEA Ratio

**Figure 10:**
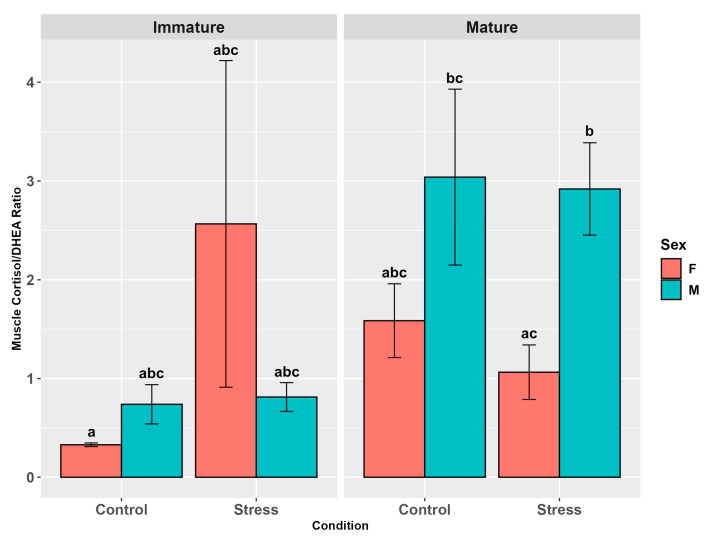
Muscle cortisol/DHEA ratio in the experimental groups by stress, sex, and maturity. Statistical analysis was performed using a multi-way ANOVA followed by Tukey post hoc test. Different letters indicate statistical differences among the groups (*p* < 0.05).

The muscle cortisol/DHEA ratio did not show any influence of stress (*p* = 0.3, Table S1c), while both sex and maturity significantly affected it (*p* < 0.001, Table S1c). Males and mature individuals showed higher levels than females (*p* < 0.01) and immature animals (*p* < 0.01), respectively (Figure 10). Interactions between sex and stress (*p* = 0.8), maturity and sex (*p* = 0.08), and maturity and stress (*p* = 0.08) did not influence the hormone ratio but the pairwise comparisons evidenced among controls higher levels in males than in females (*p* < 0.05) and in mature males than in mature female (*p* < 0.01) and immature males and females (*p* < 0.01). Mature individuals, both stressed and control, showed a higher muscle ratio than immature controls (*p* < 0.01).

The three-factor interaction (*p* < 0.05, Table S2c) revealed that stressed mature males had a higher ratio than immature female controls and stressed mature females (*p* < 0.001; *p* < 0.05, Figure 10). Mature male controls also showed a higher ratio than immature female controls (*p* < 0.001, Figure 10, Table S2c).

**Figure 11:**
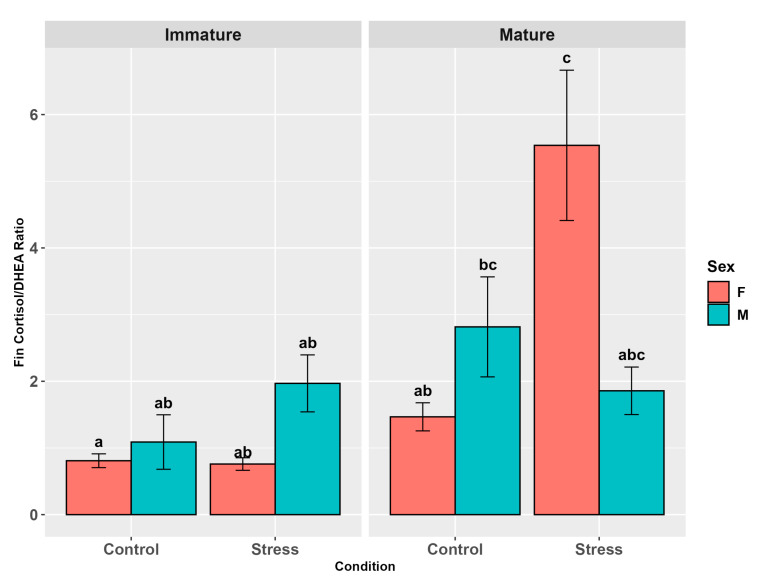
Fin cortisol/DHEA ratio in the experimental groups by stress, sex, and maturity. Statistical analysis was performed using a multi-way ANOVA followed by a Tukey post hoc test. Different letters indicate statistical differences among the groups (*p* < 0.05).

The fin cortisol/DHEA ratio was influenced by stress (*p* < 0.01) and maturity (*p* < 0.0001) but not by sex (*p* = 0.68, Table S1c). The pairwise comparisons showed a higher ratio in stressed and mature individuals than in control and immature ones, respectively (*p* < 0.01; *p* < 0.01, Table S1c). The interaction between maturity and stress was not significant (*p* = 0.53) but the pairwise comparisons showed a higher fin ratio in stressed mature individuals than in immature controls (*p* < 0.01). Maturity and sex and sex and stress interactions showed significant differences (*p* < 0.05), with both mature females and males presenting higher levels than immature females (*p* < 0.001, *p* < 0.05) and stressed females showing higher levels than the female controls (*p* < 0.01). In the three-factor interaction (*p* < 0.001, Table S2c), stressed mature females showed a higher fin ratio than the other groups (*p* < 0.01), except stressed mature males and mature male controls. The latter also showed higher levels than immature female controls (*p* < 0.05, Figure 11, Table S2c).

**Figure 12:**
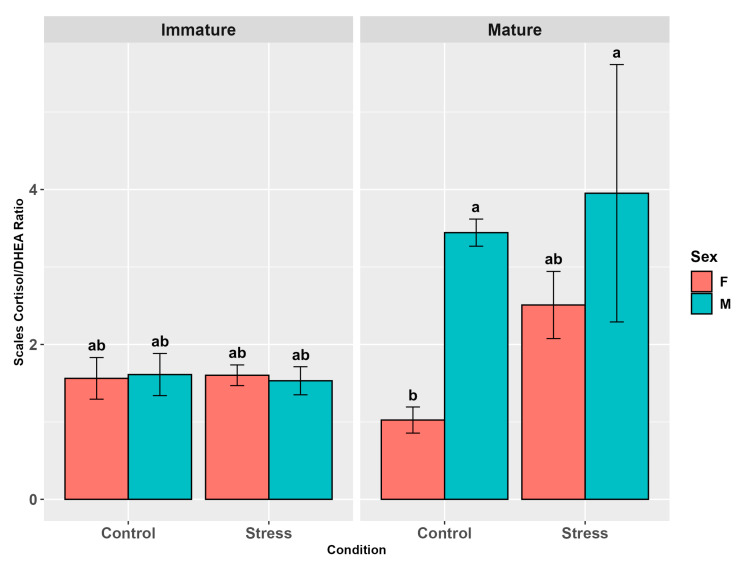
Scale cortisol/DHEA ratio in the experimental groups by stress, sex, and maturity. Statistical analysis was performed using a multi-way ANOVA followed by a Tukey post hoc test. Different letters indicate statistical differences among the groups (*p* < 0.05).

The scale cortisol/DHEA ratio did not show any influence of stress (*p* = 0.1, Table S1c) but was significantly affected by sex (*p* < 0.01) and maturity (*p* < 0.01, Table S1c), with males showing higher levels than females (*p* < 0.05) and mature individuals showing higher levels than immature ones (*p* < 0.05, Table S1c). A significant maturity and sex interaction (*p* < 0.01) showed mature males presenting higher levels than immature males, immature females, and mature females (*p* < 0.05; *p* < 0.05; *p* < 0.01, Figure 12). Maturity and stress (*p* = 0.1), sex and stress (*p* = 0.5), and the three-factor interaction (*p* = 0.05, Table S2c) did not present any differences, but in the pairwise comparisons, mature male controls and stressed mature males showed higher levels than mature female controls (*p* < 0.05; *p* < 0.01, Figure 12) and stressed males, higher levels than female controls (*p* < 0.05).

### 3.5. Correlation Among Matrices

Serum cortisol levels were correlated with those in muscle and fin, but not with those in scales or with body weight (Table S3a). Serum DHEA showed significant correlation only with the muscle (Table S3b). As for the cortisol/DHEA ratio, muscle was correlated with the fin and body weight (Tables S3c and S4).

### 3.6. Morphological Evaluation of the Gonads

**Figure 13:**
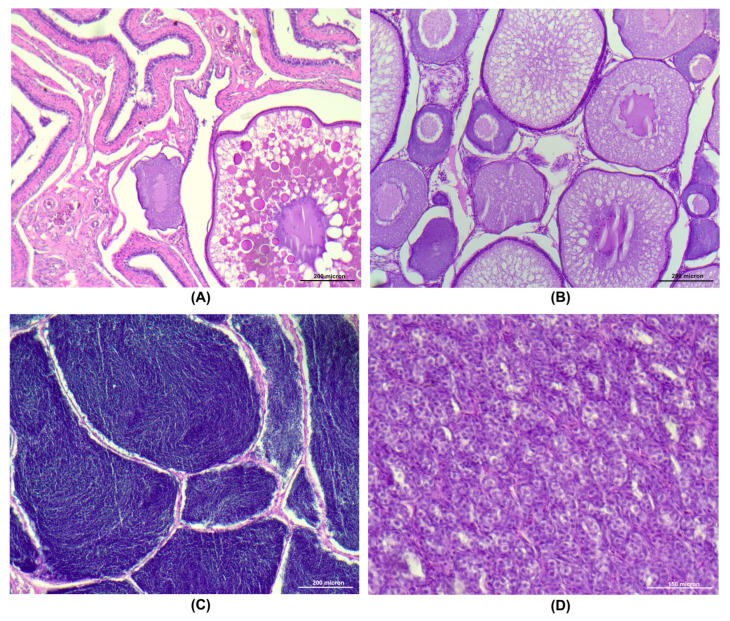
Gonadal stage of mature and immature trout. (**A**) Mature ovary with postovulatory follicles (POFs), cortical alveoli (CA), and vitellogenic oocytes; (**B**) Immature ovary with perinucleolar and CA oocytes; (**C**) Mature testis with spermatozoa filling the lobular lumens; (**D**) Immature testis with only spermatogonia. Scale bars: 200 micron (**A**–**C**); 150 micron (**D**).

The histological analysis of the gonads confirmed the maturity/immaturity status of the fish. All the mature females presented ovaries with postovulatory follicles (POFs), occupying a large part, and some cortical alveoli (CA) and/or vitellogenic oocytes (Figure 13A) and were graded as stage 5 (post ovulatory). Immature females had ovaries with perinucleolar and CA oocytes, the latter with a high amount of oil accumulation (Figure 13B) and graded as stage 1 (early development). Mature males presented the lumen of the lobules filled with spermatozoa and a very thin germinal epithelium and staged as late spermatogenic (Stage 3; Figure 13C). Lastly, the immature males were classified as undeveloped (stage 0) as their testes presented spermatogonia and spermatocytes (Figure 13D).

## 4. Discussion

This study developed RIA-based protocols to measure DHEA in rainbow trout (*Oncorhynchus mykiss*) in serum and alternative matrices (muscle, fin, scales), together with optimization of cortisol assays in the same matrices [38,41]. Hormone concentrations were assessed in adult rainbow trout in relation to acute confinement stress, sex, and maturity, with gonadal histology confirming reproductive status. As expected, circulating cortisol levels were strongly influenced by acute stress exposure and, to a lesser extent, by sex and maturity, whereas blood DHEA was unaffected by acute stress and only marginally by the other factors. In alternative matrices, hormone patterns were more variable, likely reflecting the correlation of muscle with blood and the cumulative nature of fin and scales. The cortisol/DHEA ratio reflected cortisol concentrations in blood, indicating limited usefulness under acute stress conditions, in line with findings in humans and other animals, where the ratio is primarily influenced by chronic stress [2,3]. In the alternative matrices, however, the ratios presented a more variable and less easily interpretable pattern, with marked associations with sex and maturity, possibly reflecting their potential to integrate hormonal variations over longer periods.

### 4.1. Analytic Protocols

The RIA protocols developed in this study effectively quantified DHEA and cortisol in all tested rainbow trout matrices, offering specific advantages over previous methods [30,35,36,37], particularly for scale analysis. Using diethyl ether instead of methanol simplified steroid extraction due to its faster evaporation, while the reduced sample requirement makes the method applicable not only to small or juvenile fish but also to live specimens, thereby minimizing invasiveness. This is especially relevant since scales can be collected for farm and environmental monitoring, although only limited amounts should be removed to avoid compromising their protective role.

### 4.2. Cortisol in Serum

As expected, serum cortisol significantly increased in trout subjected to 30 min pre-slaughter confinement, confirming that this treatment induced a stress response in the trout (3.36 vs. 26.82 ng/mL). Detected levels were comparable to those reported by Ruane et al. [42] in trout exposed to a longer confinement (1.5 h) but lower than those reported by Bertotto et al. [38] following a 1.5 h transport, likely reflecting the higher intensity of the latter stressor. Increases in trout blood cortisol levels due to crowding stress have been reported by others, with baseline values aligning with the present study but with post-stress variability depending on exposure conditions [43,44].

For serum cortisol, no overall interaction was detected among stress, sex, and maturity but pairwise comparisons confirmed the higher cortisol levels in all the stressed groups compared to controls. Notably, stressed immature males exhibited higher cortisol concentrations than stressed mature males, suggesting an effect of sex and maturity on the stress response amplitude. The presence of sex-related differences in cortisol response aligned with previous in vivo studies reporting lower inter-renal responsiveness in mature male trout, likely driven by sex steroid production during maturation [45,46]. In fish, as in mammals, sex steroids modulate HPI axis regulation, with estradiol generally enhancing and testosterone reducing responsiveness. In the present study, the exact stage of gonadal development in experimental animals was assessed by histological analysis and showed ready-to-spawn mature males with testes full of spermatozoa and immature males with spermatogonia with a few spermatocytes. As reported by Pottinger et al. [45,46], in trout exposed to similar stress confinement conditions, these stages are associated with high and low testosterone levels, respectively, which may explain the heightened cortisol response in immature males. Similarly to the present findings, Pottinger et al. [47] found no cortisol differences between immature sexes among the controls.

### 4.3. DHEA in Serum

Unlike cortisol, serum DHEA levels (on average 0.4–0.6 ng/mL) did not show significant changes in response to acute stress exposure, supporting the evidence that this hormone is not involved in acute stress response in trout, as also observed in goldfish by Laberge et al. [18]. Neither maturity nor the interaction among the three factors had a significant influence. However, sex and its interaction with maturity revealed an effect, with mature females showing higher levels than both immature females and unstressed mature males. This contrasts with observations in humans and other vertebrates, where DHEA levels are typically higher in males and immature females accordingly with gonadal production [48,49,50]. The reason behind these findings could be related to DHEA origin, which likely varies across species, leading to fluctuations particularly during the reproductive season, while the higher levels observed in trout females may reflect a protective role against cortisol or alternatively its function as a precursor for sex steroid synthesis. Additional studies are needed to investigate these sex-related differences and to determine whether the hormone originates mainly from the gonads, the inter-renal tissue, or even the brain.

Circulating DHEA levels observed in this study were about one order of magnitude higher than previously reported for rainbow trout and other fish species, where concentrations are often undetectable [2,30,31,51]. While cross-reactivity with DHEA-S could theoretically explain this discrepancy, the very low cross-reactivity of the antibody used (0.04%) makes it unlikely. Differences across studies are more plausibly related to the analytical method, particularly the use of commercial kits [2]. Interestingly, DHEA levels in scales were consistent with earlier findings in rainbow trout [30], suggesting that matrix-specific factors may contribute to the observed variation.

### 4.4. Cortisol in Alternative Matrices

In line with the plasma cortisol results, muscle cortisol showed a significant increase in fish exposed to acute confinement stress, supporting the close relationship between the two matrices, further confirmed by statistical correlation and consistent with our previous findings [38]. Maturity and its interaction with sex also had an effect, with higher levels observed in mature males. The interaction of all three factors revealed significant differences between immature females, stressed mature females, and both control and stressed mature males. These differences may be partly explained by intrinsic tissue factors, such as vascularization or fat content, associated with maturity and sex.

Fin cortisol did not show a significant increase directly linked to stress; however, both the three-factor interaction and the interactions between stress and sex, as well as maturity and sex, revealed significant differences, with stressed mature females showing notably higher levels than the other groups. The fin, structurally similar to scales, may serve as a better biomarker for chronic stress over extended periods [52]. Nevertheless, increased cortisol levels have also been observed after just 1.5 h of transport stress [38], as seen in the present study, indicating that further investigation is warranted. The lack of correlation with blood cortisol may reflect slower diffusion from blood to fin due to intrinsic tissue characteristics, while the significant correlation with muscle cortisol suggests that muscle may also accumulate the hormone to some extent, a hypothesis that merits further study. The elevated fin cortisol in females aligns with previous findings on the effect of sex steroids on stress response amplitude, with higher levels in mature females than in mature males. Additional studies will be necessary to better understand cortisol diffusion and accumulation dynamics in the fin.

Scale cortisol was unaffected by stress exposure, consistent with the nature of this matrix, which accumulates the hormone over time and is therefore less responsive to acute stress events. This result is further supported by the observed lack of correlation between scales and the hormone levels in blood and in the other matrices, in agreement with previous studies [30,53,54]. Among the factors, sex and maturity, as well as their interaction, significantly influenced cortisol levels, with mature males showing higher levels than both mature females and immature males. A possible explanation for this finding is the previously mentioned challenge hypothesis, whereby male competition during the reproductive season could elevate hormone levels and stress [4,5,6].

It should be noted that in the present study, scale samples were pooled from two individuals of the same group due to the limited amount of material available. This may have reduced individual variability and differences between groups; therefore, the present results should be confirmed with a larger sample size. Nevertheless, it is worth noting the detected scale cortisol levels (1–3 ng/g in the present study) are consistent with previously reported values in rainbow trout (1–2 ng/g by Kennedy et al. [30]; 0.2–1 ng/g by Carbajal et al. [53] and in other species [54,55,56]).

### 4.5. DHEA in Alternative Matrices

Muscle DHEA was significantly affected by stress exposure and sex but not by maturity. The interaction among the three factors was not significant but pairwise comparisons showed higher levels in stressed mature females than in stressed mature males and in both mature and immature male controls. These findings are compatible with the theory of a higher amplitude stress response in mature females already described in fish and other species [45,57]. DHEA concentrations in serum and muscle were significantly correlated, confirming the relation between the hormonal levels of these matrices already observed for cortisol in this and other studies [36,37,38,53].

Fin DHEA was significantly affected by stress, sex, and maturity, and the three-factor interaction, despite the absence of a significant difference, showed, at the pairwise comparisons, higher values of DHEA in fin of immature female controls than in mature female and male controls and stressed immature males. This result is compatible with its role as a sexual steroid precursor. DHEA concentrations in muscle and fin were significantly correlated, confirming the relation between the hormonal levels of these matrices already observed for cortisol in this and other studies [36,37,38,53]. No significant correlation was found between serum and fin, despite such a relationship having been previously observed for cortisol. This unexpected result may be attributed to the chemical nature of DHEA, the specific properties of the matrices, or its low circulating levels, which may have limited its detectability and distribution [30].

The scale DHEA levels were not affected by acute stress exposure nor by sex or maturity or by all the two- and three-factor interactions. Accordingly, no significant correlations were detected between scale DHEA and hormone levels in other matrices. These results align with previous studies in rainbow trout and other fish species, indicating that scales respond more to chronic than to acute stress due to their ability to accumulate hormones over time [30,53,54]. The use of pooled samples from two individuals, dictated by limited material availability, may have reduced individual variability and increased differences among groups [58]. Nonetheless, the detected scale DHEA levels (1–3 ng/g in the present study) are consistent with previously reported values in rainbow trout (0.5–1.8 ng/g by Kennedy et al. [30]; 0.4–2.5 ng/g by Carbajal et al. [53]) but further studies are needed to confirm our findings under acute stress and better understand hormone accumulation dynamics and their variation over time.

### 4.6. Cortisol/DHEA Ratio

In this study, the cortisol/DHEA ratio in blood reflected cortisol concentrations, showing higher levels in stressed trout and a sex-related effect, with mature males displaying higher values than mature females. This agrees with findings in humans and other animals under acute stress and confirms the limited usefulness of this ratio as an additional biomarker of HPI axis function beyond cortisol.

In alternative matrices, the ratio exhibited a more variable pattern, with stress effects in fin, maturity-related increases in both fin and muscle, and a trend toward significance in scales. Taken together, these findings suggest that while the blood ratio primarily reflects acute stress responses, fin, muscle, and scales may provide additional information related to sex, maturity, and potentially longer-term stress effects. Mature males consistently showed higher ratios than both immature males and mature females, further highlighting sex- and maturity-related influences.

## 5. Conclusions

This study provides new insights into the role of DHEA and cortisol in the stress response of rainbow trout, highlighting the utility of alternative matrices in hormonal monitoring. The significant rise in cortisol following acute stress confirmed the effectiveness of pre-slaughter confinement as a stressor, with fin tissue emerging as a promising minimally invasive matrix capable of reflecting this response alongside blood. Scales, by contrast, remained as expected, unaffected.

Blood DHEA levels did not vary significantly following the stressor, confirming its limited involvement in acute stress in fish. Indeed, significant increases in DHEA levels were detected in muscle and fin, suggesting these matrices to be more sensitive tools to evaluate the dynamics of this hormone. Interestingly, sex- and maturity-related trends emerged in alternative tissues. The higher DHEA levels in the fin of females and immature individuals suggest possible roles in sex steroid synthesis or cortisol metabolism. These findings align with known sex-related differences in DHEA activity in other vertebrates and point to a need for further investigation into the origin of DHEA in fish, whether it is inter-renal, gonadal, or even neural.

The cortisol/DHEA ratio in serum mirrored cortisol dynamics, indicating limited utility for acute stress assessment, whereas tissue-specific DHEA variation may reflect longer-term influences, highlighting the need for further research on its role under chronic stress.

Although blood levels still require confirmation, these findings underscore the utility of the optimized method for evaluating DHEA in alternative matrices. These matrices not only allow for minimally or non-invasive sampling and post-mortem assessment when blood is unavailable, but also appear, to some extent, more responsive than serum in detecting hormonal changes. This observation highlights the potential of these matrices in monitoring physiological states, although further research is clearly needed to better understand the timing and mechanisms of hormone transfer to each matrix. Additional research is needed to explore hormone dynamics under chronic stress conditions and to clarify the role of sex and reproductive status.

## Figures and Tables

**Table 1:** Experimental groups: sex, sexual maturity, stress exposure, and total body weight. A multi-way ANOVA was conducted followed by a Tukey post hoc test. Different letters indicate significant differences among the groups.

Group	Sex	Maturity	Stress	Weight (g)	*p* < 0.05
1	Female	Mature	Control	975.77 ± 36.98	a
2	Female	Mature	Stress	1015.38 ± 40.33	a
3	Female	Immature	Control	1280.00 ± 70.67	bc
4	Female	Immature	Stress	1419.17 ± 63.89	b
5	Male	Mature	Control	1053.33 ± 57.23	ac
6	Male	Mature	Stress	955.83 ± 56.78	a
7	Male	Immature	Control	1461.67 ± 28.41	b
8	Male	Immature	Stress	1326.67 ± 54.74	b

**Table 2:** R^2^, extraction yield, (EY; *n* = 8 replicates), intra-assay, and inter-assay values for precision and accuracy for the determination of cortisol and DHEA in the different matrices.

Cortisol				
Matrix	R^2^	EY%	Intra	Inter
Serum	0.9983	85	4.26	2.69
Muscle	0.9948	80	7.68	5.49
Fin	0.9943	84	5.81	4.85
Scales	0.9997	82	5.06	3.67
**DHEA**				
Matrix	R^2^	EY%	Intra	Inter
Serum	0.9983	74	5.55	2.26
Muscle	0.9948	72	8.33	7.54
Fin	0.9943	71	4.94	3.77
Scales	0.9997	79	8.73	5.19

## Data Availability

The datasets generated during and/or analyzed during the current study are available from the corresponding author on reasonable request.

## Supplementary Materials

The following supporting information can be downloaded at: https://www.mdpi.com/article/10.3390/ani15182710/s1, Table S1a–c: Hormones concentration per matrix by stress, sex, and sexual maturity; Table S2a–c: Hormones concentration per group; Table S3a–c: Pearson correlation coefficient between matrices; Table S4: Pearson correlation coefficient between weight and matrices.

## Abbreviations

The following abbreviations are used in this manuscript:

DHEA	Dehydroepiandrosterone
DHEAS	Dehydroepiandrosterone Sulfate
P450c17	17 alphahydroxylase/17,20 lyase
11βHSD2	11betaHydroxysteroid Dehydrogenase type 2
HPA	Hypothalamic–Pituitary–Adrenal axis
HPI	Hypothalamic–Pituitary–Inter-renal axis
ACTH	Adrenocorticotropic Hormone
RIA	RadioImmunoAssay
PBS	Phosphate-Buffered Saline
BSA	Bovine Serum Albumin
CA	Cortical Alveoli
POFs	Post-Ovulatory Follicles
