# Supplementary material for: DHEA and Cortisol in Rainbow Trout (Oncorhynchus mykiss): Effect of Sex, Sexual Maturity, and Acute Stress Exposure
Source: Animals (Basel). 2025 Sep 16;15(18):2710. doi: 10.3390/ani15182710 (PMC12466356; doi:10.3390/ani15182710)
Supplement: Supplementary file 1 [file animals-15-02710-s001.zip › animals-3834192-supplementary.pdf]

Supplementary Materials

Table S1.

Average levels of cortisol (a), DHEA (b) and Cortisol/DHEA ratio (c) in the different experimental groups per factors and in the different matrices.

Table a)

Cortisol						
Condition	**Control**	**Stress**	**Female**	**Male**	**Mature**	**Immature**
**Serum** (ng/ml)	3.36 ± 1.02	26.82 ± 4.91	14.55 ± 4.2	15.66 ± 5.79	13.95 ± 3.9	16.28 ± 5.97
**Muscle** (ng/g)	0.68 ± 0.26	1.1 ± 0.41	1.02 ± 0.45	0.79 ± 0.27	1.27 ± 0.38	0.46 ± 0.29
**Fin** (ng/g)	1.25 ± 0.23	2.09 ± 0.66	1.96 ± 0.64	1.28 ± 0.3	2.23 ± 0.68	1.06 ± 0.17
**Scale** (ng/g)	1.74 ± 0.27	2.16 ± 0.36	1.61 ± 0.21	2.36 ± 0.41	2.35 ± 0.42	1.53 ± 0.13

Table b)

DHEA						
Condition	**Control**	**Stress**	**Female**	**Male**	**Mature**	**Immature**
**Serum** (ng/ml)	0.52 ± 0.11	0.6 ± 0.15	0.65 ± 0.17	0.47 ± 0.09	0.61 ± 0.15	0.51 ± 0.13
**Muscle** (ng/g)	0.44 ± 0.07	0.75 ± 0.3	0.79 ± 0.31	0.4 ± 0.09	0.71 ± 0.31	0.46 ± 0.07
**Fin** (ng/g)	0.86 ± 0.14	0.7 ± 0.14	0.87 ± 0.15	0.67 ± 0.13	0.63 ± 0.12	0.93 ± 0.15
**Scale** (ng/g)	0.83 ± 0.06	0.76 ± 0.04	0.83 ± 0.06	0.76 ± 0.04	0.82 ± 0.05	0.78 ± 0.06

Table c)

Ratio Cortisol/DHEA						
Condition	**Control**	**Stress**	**Female**	**Male**	**Mature**	**Immature**
**Serum**	5.49 ± 1.48	43.83 ± 7.17	23.33 ± 7.64	28.17 ± 8.05	24.01 ± 6.45	27.5 ± 9.05
**Muscle**	1.41 ± 0.54	1.74 ± 0.59	1.22 ± 0.60	1.88 ± 0.6	2.17 ± 0.59	0.94 ± 0.53
**Fin**	1.53 ± 0.44	2.75 ± 0.83	2.32 ± 0.87	1.94 ± 0.51	3.03 ± 0.86	1.18 ± 0.33
**Scale**	1.72 ± 0.27	2.3 ± 0.42	1.6 ± 0.22	2.48 ± 0.47	2.4 ± 0.48	1.58 ± 0.11

Table S2.

Average levels of cortisol (a), DHEA (b) and Cortisol/DHEA ratio (c) in the different experimental groups and in the different matrices.

Table a)

Cortisol				
	**Serum** (ng/ml)	**Muscle** (ng/g)	**Fin** (ng/g)	**Scales** (ng/g)
**Female Mature**	5.36 ± 1.33	1.07 ± 0.24	1 ± 0.16	1.03 ± 0.1
**Female Mature Stressed**	27.79 ± 4.62	1.5 ± 0.54	4.49 ± 0.91	2.45 ± 0.26
**Female Immature**	1.57 ± 0.32	0.19 ± 0.002	1.24 ± 0.16	1.6 ± 0.13
**Female Immature Stressed**	23.12 ± 2.17	1.31 ± 0.69	0.95 ± 0.16	1.66 ± 0.11
**Male Mature**	4.64 ± 1.19	1.2 ± 0.39	1.67 ± 0.39	3.45 ± 0.16
**Male Mature Stressed**	17.55 ± 2.44	1.25 ± 0.24	1.45 ± 0.37	3.74 ± 0.59
**Male Immature**	1.7 ± 0.61	0.35 ± 0.16	1.1 ± 0.15	1.67 ± 0.17
**Male Immature Stressed**	38.74 ± 7.6	0.38 ± 0.07	0.93 ± 0.2	1.19 ± 0.07

Table b)

DHEA				
	**Serum** (ng/ml)	**Muscle** (ng/g)	**Fin** (ng/g)	**Scales** (ng/g)
**Female Mature**	0.71 ± 0.08	0.45 ± 0.04	0.55 ± 0.03	0.84 ± 0.07
**Female Mature Stressed**	0.88 ± 0.24	1.49 ± 0.55	0.8 ± 0.18	0.78 ± 0.03
**Female Immature**	0.54 ± 0.2	0.48 ± 0.03	1.3 ± 0.14	0.86 ± 0.1
**Female Immature Stressed**	0.39 ± 0.02	0.65 ± 0.11	0.91 ± 0.12	0.83 ± 0.04
**Male Mature**	0.38 ± 0.04	0.46 ± 0.14	0.5 ± 0.04	0.8 ± 0.05
**Male Mature Stressed**	0.37 ± 0.03	0.4 ± 0.09	0.67 ± 0.14	0.83 ± 0.05
**Male Immature**	0.38 ± 0.05	0.35 ± 0.05	1.1 ± 0.16	0.82 ± 0.02
**Male Immature Stressed**	0.7 ± 0.14	0.38 ± 0.02	0.41 ± 0.06	0.62 ± 0.02

Table c)

Ratio Cortisol/DHEA				
	**Serum**	**Muscle**	**Fin**	**Scales**
**Female Mature**	5.5 ± 1.02	1.59 ± 0.32	1.47 ± 0.21	1.02 ± 0.12
**Female Mature Stressed**	35.11 ± 6.58	1.06 ± 0.29	5.54 ± 1.17	2.51 ± 0.25
**Female Immature**	2.95 ± 0.49	0.32 ± 0.01	0.81 ± 0.1	1.56 ± 0.16
**Female Immature Stressed**	50.23 ± 7.97	2.57 ± 1.26	0.76 ± 0.08	1.6 ± 0.08
**Male Mature**	11.36 ± 2.25	3.04 ± 0.89	2.82 ± 0.72	3.44 ± 0.09
**Male Mature Stressed**	41.45 ± 6.21	2.92 ± 0.47	1.86 ± 0.33	3.95 ± 0.83
**Male Immature**	2.98 ± 0.78	0.74 ± 0.2	1.09 ± 0.39	1.61 ± 0.16
**Male Immature Stressed**	49.61 ± 8.85	0.81 ± 0.15	1.97 ± 0.43	1.53 ± 0.1

Table S3.
Pearson correlation coefficient between matrices and p value, NA = not available, * $p < 0.05$, ** $p < 0.01$, *** $p < 0.001$, ns = not significant

Table a)

Cortisol								
	**Serum**	**p value**	**Muscle**	**p value**	**Fin**	**p value**	**Scales**	**p value**
**Serum**	1	NA	0.30	**	0.28	**	-0.02	ns
**Muscle**	0.30	**	1	NA	0.49	***	0.05	ns
**Fin**	0.28	**	0.49	***	1	NA	-0.03	ns
**Scales**	-0.02	ns	0.05	ns	-0.03	ns	1	NA

Table b)

DHEA								
	**Serum**	**p value**	**Muscle**	**p value**	**Fin**	**p value**	**Scales**	**p value**
**Serum**	1	NA	0.32	**	0.01	ns	-0.12	ns
**Muscle**	0.32	**	1	NA	0.28	**	0.05	ns
**Fin**	0.01	ns	0.28	**	1	NA	0.15	ns
**Scales**	-0.12	ns	0.05	ns	0.15	ns	1	NA

Table c)

Ratio Cortisol/DHEA								
	**Serum**	**p value**	**Muscle**	**p value**	**Fin**	**p value**	**Scales**	**p value**
**Serum**	1	NA	0.18	ns	0.07	ns	0.08	ns
**Muscle**	0.18	ns	1	NA	0.27	*	0.33	ns
**Fin**	0.07	ns	0.27	*	1	NA	0.05	ns
**Scales**	0.08	ns	0.33	ns	0.05	ns	1	NA

Table S4.
Pearson correlation coefficient between weight and matrices and p value, ** $p < 0.01$, *** $p < 0.001$, ns = not significant

	**Cortisol**		**DHEA**		**Ratio Cortisol/DHEA**	
	**Weight**	**p value**	**Weight**	**p value**	**Weight**	**p value**
**Serum**	-0.11	ns	-0.07	ns	-0.010	ns

**Muscle**	-0.34	***	-0.14	ns	-0.271	**
**Fin**	-0.22	**	0.29	**	-0.334	***
**Scales**	-0.11	ns	-0.02	ns	-0.101	ns
